# Characterizing and TRAPing a Social Stress-Activated Neuronal Ensemble in the Ventral Tegmental Area

**DOI:** 10.3389/fnbeh.2022.936087

**Published:** 2022-07-08

**Authors:** Ioannis Koutlas, Louisa E. Linders, Stef E. van der Starre, Inge G. Wolterink-Donselaar, Roger A. H. Adan, Frank J. Meye

**Affiliations:** Department of Translational Neuroscience, Brain Center, University Medical Center Utrecht, Utrecht University, Utrecht, Netherlands

**Keywords:** ventral tegmental area, social stress, neuronal ensemble, dopamine, TRAP2, c-Fos, excitability characterizing a VTA social stress ensemble

## Abstract

Social stress is a major contributor to neuropsychiatric issues such as depression, substance abuse and eating disorders. The ventral tegmental area (VTA) is involved in the effects of stress on cognitive and emotional processes perturbed in these disorders. However, the VTA is a cellularly heterogeneous brain area and it remains unclear which of its neuronal populations make up the social stress-sensitive ensemble. The current study characterizes the molecular, topographical and functional properties of VTA social stress-activated cells. First, we used immunohistochemical analysis of Fos protein, a marker of recent increased neuronal activity, to show that acute social stress activates a mainly neuronal ensemble in the VTA (VTA_Social stress_ neurons). Topographical analysis showed that this ensemble, which comprises ∼11% of all VTA neurons, occurs across VTA subregions. Further analysis showed that approximately half of the VTA_Social stress_ neurons express the dopamine synthesis rate-limiting enzyme tyrosine hydroxylase (TH). In a minority of cases this occurred with coexpression of vesicular glutamate transporter 2 (Vglut2). Also part of the ensemble were VTA cells expressing just Vglut2 without TH, and cells expressing the vesicular GABA transporter (VGAT) without TH. Next, using targeted recombination in active populations (TRAP2), we showed that VTA_Social stress_ neurons can be permanently tagged and made tractable for future functional investigations. Using a combination of TRAP2 and patch-clamp electrophysiology we demonstrate that VTA_Social stress_ neurons exhibit higher excitability than their non-TRAPed neighbor cells. Overall, this study shows that acute social stress activates an ensemble of neurons throughout the VTA, comprising distinct molecular identities, and with specific electrophysiological features. It also identifies TRAP2 as a tool to make this ensemble tractable for future functional studies.

## Introduction

Stress can trigger the intake of rewards such as high-caloric palatable food and recreational drugs (Adam and Epel, 2007; Sinha and Jastreboff, 2013; Yau and Potenza, 2013). Accordingly, in vulnerable individuals stress can contribute to the development of excess weight (Dallman, 2010), and to the continuous relapse occurring in psychiatric disorders such as bulimia nervosa, binge eating disorder and drug addiction (Adam and Epel, 2007; Sinha and Jastreboff, 2013). How stressful experiences affect the neural circuits that underlie reward processing remains an important question.

An interesting candidate hub region for the effects of stress on reward processing is the ventral tegmental area (VTA) in the midbrain (Meye and Adan, 2014; Holly and Miczek, 2016; Morales and Margolis, 2017). The VTA is a heterogeneous region, both in terms of its cell type composition and its connectivity. The VTA contains dopaminergic, GABAergic and glutamatergic neurons, but also several neurons that corelease such neurotransmitters (Morales and Margolis, 2017). VTA neurons innervate various structures, including the nucleus accumbens (NAc) and prefrontal cortex (PFC). An extensive literature links the activity of VTA dopamine neurons to processes of salience encoding and reward seeking (Bromberg-Martin et al., 2010; Cohen et al., 2012; Meye and Adan, 2014; Morales and Margolis, 2017; Mohebi et al., 2019). The response of many VTA dopamine neurons to unexpected rewards, or their cues, is increased activity. In contrast, unexpected aversive experiences, or their cues, drive acute inhibition of their activity (Cohen et al., 2012; Meye and Adan, 2014; Morales and Margolis, 2017; Mohebi et al., 2019). Importantly however, a variety of VTA neuron types also have been shown to be stimulated by aversive experiences (Brischoux et al., 2009; Bromberg-Martin et al., 2010; Lammel et al., 2014; Holly and Miczek, 2016; Morales and Margolis, 2017).

Ventral tegmental area dopamine output has been implicated in the encoding of aversive stimuli. In particular microdialysis and voltametric studies have described that stressful events trigger release of dopamine in the PFC, but also in the NAc (Holly and Miczek, 2016; Vander Weele et al., 2019). Within the NAc there is likely a strong heterogeneity across subterritories in this regard. For instance, electric shocks, and their predictive cues, increase dopaminergic output in the NAc ventral medial shell, while suppressing it in the NAc lateral shell (Jong et al., 2019). Aversion-driven firing has also been reported at the level of VTA somata. Using *in vivo* electrophysiological recordings, a subset of VTA dopamine neurons were shown to have acute increased activity in response to electrical shock or to air puffs and their cues (Brischoux et al., 2009; Matsumoto and Hikosaka, 2009; Cohen et al., 2012). Also fiber photometric calcium recordings showed that there are excitatory responses of (a subpopulation of) VTA dopamine neurons in response to aversive stimuli like a male intruder (Moriya et al., 2018), or electrical shocks (Cai et al., 2020). Also non-dopaminergic VTA neurons are responsive to aversive stimuli. *In vivo* electrophysiological recordings demonstrated that putative VTA GABA neurons can be excited by aversive stimuli like shocks (Tan et al., 2012) or air puffs and their predictive cues (Cohen et al., 2012). Accordingly, recent fiber photometric studies showed that VTA GABA neurons exhibit calcium transients in response to shocks (Root et al., 2020). The same holds true for VTA glutamate neurons (Root et al., 2020), which are also excited by other types of aversive stimuli, like a threatening looming shadow (Barbano et al., 2020). Combinatorial GABA/glutamate VTA neurons are also reactive to aversive shocks (Root et al., 2020). Overall, these data suggest that the VTA neuronal ensemble that is stimulated by aversive experiences comprises cell types with different neurotransmitters.

Notably, aversive stimuli differ considerably in their properties between each other (Nakamura et al., 2022). Many of the stimuli used to study VTA encoding of aversive experiences have consisted of brief transient stimuli (e.g., electric shocks, air puffs), rather than more complex stressors. Social stress is a naturalistic stressor which can contribute to the development of excess food intake and weight gain (Razzoli et al., 2015), to exacerbated drug-seeking behavior (Holly and Miczek, 2016; Newman et al., 2018) and to the development of depressive-like phenotypes (Chaudhury et al., 2013; Friedman et al., 2014). Social stress engages the VTA (Tanaka et al., 2012; Chaudhury et al., 2013; Morel et al., 2017), but it remains largely unclear what the molecular identity and functional properties are of the VTA social stress-sensitive (VTA_Social stress_) ensemble. Here we address these issues by leveraging stress-driven Fos activity, both immunohistologically and by using a transgenic line in which c-Fos promotor activity results in conditional nuclear entry of Cre recombinase (Targeted Recombination in Active Populations; TRAP2) (DeNardo et al., 2019). We demonstrate the presence of a molecularly heterogeneous VTA_Social stress_ ensemble that comprises approximately 11% of the VTA and is distributed across its subregions. Furthermore, we observe that these neurons exhibit an electrophysiological profile that is distinct from its neighboring cells. Overall this study characterizes the VTA_Social stress_ ensemble, and shows how it can be made tractable using the TRAP2 strategy for future functional studies.

## Materials and Methods

### Animals

Adult male mice (25–35 g, >8 weeks) were used in all experiments. C57Bl6J (Jax #664), heterozygous vesicular GABA transporter-Cre (VGAT-Cre; Jax #016962), heterozygous vesicular glutamate transporter 2-Cre (Vglut2-Cre; Jax #28863), heterozygous TRAP2 (Jax #030323), heterozygous mice Ai14 tdTomato reporter (Jax #007914), and double heterozygous TRAP2xAi14 offspring were bred in house, but originated from the Jackson Laboratory. For the social stress paradigm, proven breeder Swiss-CD1 mice were purchased from Janvier (France) and were used as aggressors. All mice (except Swiss-CD1 mice) were group-housed (2–4 per cage) in a temperature- and humidity-controlled room (22 ± 2°C and 60–65% respectively) under a 12 h light/dark cycle (lights on at 7am) with ad libitum access to water and standard laboratory chow [Special Diet Services [SDS], product code CRM(E)]. All experiments were approved by the Animal Ethics Committee of Utrecht University, and were conducted in agreement with Dutch law (Wet op de Dierproeven, 2014) and European regulations (Guideline 86/609/EEC).

### Stereotactic Injections

Mice were anesthetized with ketamine (75 mg/kg i.p.; Narketan, Vetoquinol) and dexmedetomidine (1 mg/kg i.p.; dexdomitor, Vetoquinol). For local anaesthesia, Lidocaine (0.1 ml; 10% in saline; B. Braun) was topically applied under the skin on the skull before incision. Eye ointment cream (CAF, Ceva Sante Animale B.V., Naaldwijk, Netherlands) was applied before surgery. Mice were mounted on a stereotactic frame (UNO B.V., Zevenaar, Netherlands, Model 68U801 or 68U025) and kept on a heating pad (33°C) during surgery. Injections were done using a 31G metal needle (Coopers Needleworks, Birmingham, United Kingdom) attached to a 10 μl Hamilton syringe (model 801RN) via flexible tubing (PE10, 0.28 mm ID, 0.61 mm OD, Portex, Keene, NH, United States). The Hamilton syringe was controlled by an automated pump (UNO B.V., Zevenaar, Netherlands, -model 220). Animals were bilaterally injected in the VTA (Relative to Bregma: 3.2 mm posterior, 1.6 mm lateral, and 4.9 mm ventral) at a 15^o^ angle with 0.3 μl of AAV per hemisphere at a rate of 0.1 μl/min. rAAV-hSyn-DIO-mCherry (4.2 × 10^12^ gc/ml; UNC Vector Core) or rAAV-EF1a-DIO-mCherry (3.6 × 10^12^ gc/ml; UNC Vector Core) was injected in the VTA of VGAT-Cre or Vglut2-Cre mice to visualize VTA GABAergic and glutamatergic subpopulations respectively. After infusion of the viral volume, needles were left in place for an additional 6.5 min. Then they were moved 50 μm dorsally and completely retracted 30 s later. The skin was subsequently sutured (V926H, 6/0, VICRYL, Ethicon). Mice were then subcutaneously injected with atipamezole (50 mg/kg; Atipam, Dechra), carprofen (5 mg/kg, Carporal) and 1 ml of saline and were left to recover on a heating pad at 36°C. Carprofen (0.025 mg/l) was provided in drinking water for a week after surgery. Animals were solitarily housed for 3 days post-surgery. Mice were left to recover for at least 4 weeks prior to subsequent behavioral manipulations. VGAT-Cre (*n* = 6) and Vglut2-Cre (*n* = 1) mice with misplaced injections were excluded from the final analysis.

### Social Stress Paradigm

Swiss-CD1 male mice were housed individually in a Makrolon cage (type IV, Tecniplast, Buguggiate, Italy). An intruder experimental mouse was introduced in the cage between 8:00 – 10:30 am. Fighting was tracked live and animals were allowed to fight for an accumulated 20 s. Next, they were separated by a perforated transparent splitter that prevented physical, but allowed sensory interaction for the remaining time of the experiment. For Fos immunohistochemical experiments, animals remained in the CD1 aggressor cage until perfusion (90 min later). Control animals were placed in a Makrolon cage with a novel C57Bl6J male cage mate separated by a transparent splitter and were perfused 90 min later.

For TRAP2xAi14 experiments, animals were solitarily housed 7 days before the stress episode. During this period, mice were habituated to intraperitoneal (i.p.) injections by receiving a saline injection i.p., 4 and 5 days after the start of their solitary housing. During the social stress paradigm, TRAP2xAi14 mice were put in the resident cage and were allowed to fight for an accumulated total of 20 s. Afterward they remained in the resident cage, separated by a perforated transparent splitter until they received an injection (i.p.) of 4-hydroxytamoxifen (4-OHT) at a dose of 25 mg/kg body weight. The 4-OHT (Sigma-Aldrich Chemie N.V, Zwijndrecht, Netherlands, H6278) was dissolved in an aqueous solution as described before (Ye et al., 2016). The final solution contained 2.5 mg/ml 4-OHT, dissolved in 5% DMSO, 1% Tween-80 and saline. As indicated in the Results section, depending on exact experimental conditions, the 4-OHT injection occurred 1, 2, 3, or 4 h after the start of the social stress episode. To ensure 4-OHT-dependency of c-Fos-driven recombination, one TRAPxAi14 control group received social stress but no i.p. 4-OHT injection. Otherwise, regular control animals were again paired with a novel cage mate (TRAP2xAi14 mice), without experiencing social defeat. These mice were also separated by a transparent splitter for an equal amount of time as the respective stress group (1–4 h), and also received 4-OHT injections. For TRAP2xAi14 experiments, all mice were placed back in their home cage (solitarily housed) and were transcardially perfused 7 days later. Instead, TRAP2xAi14 animals that were exposed to a second social stress episode or that were used for electrophysiological experiments were solitarily housed for 3 or 4 weeks after the initial stress episode, respectively.

### Immunohistochemistry

Animals were transcardially perfused with 4% paraformaldehyde (PFA) and brains were post-fixated in 4% PFA at 4°C for 24 h. Next, they were transferred to a 30% sucrose solution in PBS where they remained for another 48–72 h. Coronal sections of 35 μm were made with a cryostat (CM1950, Leica, Netherlands). Slices were first washed 4 × 10 min in PBS followed by incubation in blocking solution [5% normal goat serum (NGS), 2.5% bovine serum albumin, 0.2% Triton X-100 in PBS] for 1 h. Subsequently, slices were moved to the primary antibody solution consisting of rabbit-anti-c-Fos (1:1000; Cell Signaling Technology, 2250s, Leiden, Netherlands) and mouse-anti-TH (1:1000; Sigma-Aldrich Chemie N.V, MAB318, Zwijndrecht, Netherlands) or mouse-anti-NeuN (1:1000; Abcam, ab104224, Waltham, MA, United States) in blocking solution overnight. The following day slices were washed 4 × 10 min followed by 2 h incubation in secondary antibody solution containing goat-anti-rabbit Alexa Fluor 488 (1:500; Abcam, ab150077, Waltham, MA, United States), and goat-anti-mouse Alexa Fluor 647 (1:500; Thermo Fischer Scientific, A-21236, Waltham, MA, United States). Slices then were moved to DAPI (0.5 μg/ml; Biotium brand, VWR, #89139-054, Netherlands) for 5 min followed by 2 × 10 min washes in PBS. Finally, slices were mounted on glass slides using 0.2% gelatin in PBS and coverslipped using FluorSave (Millipore, 345789, Amsterdam, Netherlands).

Biocytin-filled sections from patch-clamp experiments were washed 4 × 30 min in PBS followed by incubation in blocking solution (10% NGS, 0.5% Triton X-100) for 5 h in room temperature. Next, slices were incubated in mouse-anti-TH (1:1000; Sigma-Aldrich Chemie N.V, MAB318, Zwijndrecht, Netherlands) and 488-conjugated streptavidin (1:500; Thermo Fischer Scientific, S11223, Waltham, MA, United States) in 2% NGS, 0.4% Triton X-100 in PBS for 48 h at 4°C. Next, slices were washed in PBS 4 × 30 min followed by incubation in goat-anti-mouse Alexa Fluor647 (1:500; Thermo Fischer Scientific, A-21236, Waltham, MA, United States) for 5 h in room temperature. Finally, slices were washed in PBS 4 × 30 min, mounted on glass slides using 0.2% gelatin in PBS and coverslipped using FluorSave (Millipore, 345789, Amsterdam, Netherlands).

### Cell Counting and Topography

For cell counting in the VTA, 4-6 images of a hemisection of the VTA were acquired using a wide-field epifluorescence microscope (AX10, Zeiss). Specifically a multi-channel image was acquired where the TH staining along with other anatomical landmarks such as the interpenducular nucleus (IPN), substantia nigra compact part (SNc), the medial lemniscus (ml) and the red nucleus (RN) were used to determine the boundaries of the VTA. Fiji software^1^ was used to manually draw the boundaries of VTA hemisections using TH staining outlines. Next, cells expressing Fos or tdTomato were manually counted by an experimenter blinded to the experimental conditions. Cell density was then expressed as the number of Fos or tdTomato cells over the area of the given region of interest. For co-localization analysis 4–6 z-stacks were acquired using a confocal microscope (LSM 880, Zeiss). Also in this case, the TH staining along with other anatomical landmarks were used to determine the VTA hemisection boundaries and cells were manually counted by an experimenter blinded to the conditions of the experiment.

For topographical analysis Fos^+^ or stress-TRAPed cells were manually selected using the Fiji multipoint tool. For each slice a reference point along the middle of the midline of VTA was selected based on the TH staining channel. The XY coordinates of these cells were acquired and subsequently plotted against this reference point. A correction was made in case the slice was mounted at an angle. Next, based on the TH staining channel and the Paxinos brain atlas (2nd edition; Academic Press) the approximate anterior-posterior (AP) coordinate of each VTA slice was estimated, and categorized as rostral (–2.92 < –3.20 mm), Middle (–3.20 < –3.50 mm) or Caudal (–3.50 < –3.90 mm) VTA. For each animal (C57Bl6J, *n* = 7; TRAP2xAi14, *n* = 6) 6 VTA slices (2 per AP zone) were included. Subsequently, XY coordinates were grouped together per AP zone and the frequency distribution of X (mediolateral) and Y (dorsoventral) was plotted in bins of 100 microns.

In order to look for possible mediolateral or dorsoventral gradients, we compared the observed X and Y frequency distributions with topographical distributions acquired from randomly generated points within model VTA topographical boundaries. We used a custom-made MATLAB script to randomly generate points within a model rostral (–3.08 mm), middle (–3.40 mm), and caudal (–3.80 mm) VTA. First, the general outline of a VTA hemisection was manually drawn with points in Fiji. Next the XY coordinates of these points were acquired and used in the MATLAB script for it to generate a polygon with the given shape. Next a predefined number of points, equal to the observed number of Fos^+^ neurons per AP zone, was randomly generated. Frequency distributions of the randomly generated points were superimposed over the observed distributions and statistically compared.

For subregion analysis, the parabrachial pigmented area (PBP), paranigral nucleus (PN), interfascicular nucleus (IF) and the rostral linear nucleus (RLi) were included. The borders of VTA subregions were determined based on the representative Paxinos Brain Atlas images and the respective XY coordinates were acquired using Fiji. A custom-made MATLAB script was then used to generate 2-D polygons given the XY coordinates of the respective subregions that were superimposed on a single 2-D plane (Supplementary Figure 2B). Next, the XY coordinates of the Fos^+^/TRAPed cells were plotted in the same plane and the number of neurons that was located within each polygon (VTA subregion) was automatically counted with a custom-made MATLAB script. Two sets of random points of size n_1_ and n_2_ within the VTA geometric boundaries were generated with *n*_1_ = total number of observed Fos^+^ cells and *n*_2_ = total number of observed TRAPed cells. Subsequently, the same custom-made MATLAB script was used on these two sets to determine the proportion of neurons per subregion expected by chance. Finally, the observed and expected subregional distributions were statistically compared.

### Patch-Clamp Electrophysiology

Animals were anesthetized with isoflurane (Zoetis, Leatherhead, United Kingdom) between 8.30 a.m. and 10 a.m. and were then rapidly decapitated. Coronal brain slices of 250 μm were cut on a vibratome (1200 VTs, Leica, Rijswijk, Netherlands) in ice-cold carbogenated (95% O2, 5% CO2) cutting solution, containing (in mM) choline chloride 92; ascorbic acid 10; CaCl2 0.5; glucose 25; HEPES 20; KCl 2.5; *N*-Acetyl L Cysteine 3.1; NaHCO3 25; NaH2PO4 1.2; *N*-metyhl D glucamine (NMDG) 29; MgCl2 7; sodium pyruvate 3; Thiourea 2. Subsequently slices were transferred for 5 min to warmed solution (34 °C) of identical composition, before they were stored at room temperature in carbogenated incubation medium containing (in mM): NaCl 92; KCl 2.5; ascorbic acid 3; CaCl2 2; glucose 25; HEPES 20; NaHCO3 20; NaH2PO4 1.2; MgCl2 2; sodium pyruvate 3 and Thiourea 2. For recordings, slices were immersed in artificial cerebrospinal fluid (ACSF) containing (in mM): NaCl 124; KCl 2.5; glucose 11; HEPES 5; NaHCO3 26; NaH2PO4 1; CaCl2 2.5; MgCl2 1.3 and were continuously superfused at a flow rate of 2.5 ml/min at 32° C (Montalban et al., 2022).

Neurons in the VTA were patch-clamped using borosilicate glass pipettes (2.7–4 MΩ; glass capillaries, GC150-10, Harvard apparatus, Cambridge, United Kingdom), under a TH4-200 Olympus microscope (Olympus, Rungis, France). Recordings were made in current clamp in a potassium gluconate based internal solution containing (in mM), potassium gluconate 139; HEPES 10; EGTA 0.2; creatine phosphate 10; KCl 5; Na2ATP 4; Na3GTP 0.3; MgCl2 2 with 0.05% Biocytin. Signal was amplified, low-pass filtered at 2.9 kHz with a 4-pole Bessel filter, and digitized at 20 kHz with an EPC10 dual patch-clamp USB amplifier (HEKA Elektronik GmbH, Lambrecht, DE). Data were acquired using PatchMaster v2x90.2 software. To assess the excitability profile and biophysical properties, patched neurons were subjected to 17 subsequent current steps from 0 pA (one every 10 s) of 800 ms length, starting from –150 pA, with a 25 pA increasing increment/step. Data were subsequently analyzed in Igor pro 8 (Wavemetrics, Tigard, OR, United States). The rheobase was identified as the minimal current to elicit the first action potential during the 800 ms window. The action potential threshold was determined by differentiating the waveform eliciting the first action potential, and finding the corresponding voltage level (in the non-differentiated waveform) at which a 20 mV/ms value was surpassed. The resting membrane potential was determined at the 0 pA injection step, in case of a non-spontaneously firing neuron. The capacitance of the cell was determined using the HEKA amplifier read-out, using a 4 mV hyperpolarizing step in voltage-clamp. The voltage sag was calculated as the delta voltage between the minimal voltage deflection versus the steady-state voltage in response to a –150 pA current injection. The membrane resistance was calculated according to Ohm’s law during negative current injection steps not eliciting active conductances. Identifying a patched cell as being part of either lateral or medial VTA was done by post-hoc evaluation of images for recordings of the VTA slice with the patch pipette in it, separating the VTA in a medial and a lateral half, and allocating the patched neuron accordingly, while being blind to the electrophysiological profile.

### Statistical Analysis

Data were analyzed using GraphPad Prism 9.3.1 (San Diego, CA, United States), SPSS 28 (IBM, Armonk, NY, United States) and MATLAB (Mathworks, Natick, MA, United States). Animals were randomly assigned to experimental conditions. To test whether our data were sufficiently modeled by a normal distribution, the Kolmogorov–Smirnov test was performed. To test for differences in Fos expression between control and stress groups, two-tailed unpaired *t*-tests were used. For topographical comparisons data from all animals were pooled together and a correction for sample size was made (by acquiring a random sample of size n/number of animals used in the analysis). To determine whether the topographical distribution of Fos^+^ cells differed from a randomly generated distribution, Wald-Wolfowitz runs tests were performed with a corrected alpha (α = 0.0083) to accommodate for multiple comparisons. To test for differences in the topographical distribution of Fos^+^ and TRAPed neurons within VTA subregions, Chi-square tests and a Two-way ANOVA test were used. To test for differences in TRAPed cell density at different time points of 4-OHT injection, a non-parametric Kruskal–Wallis test with post-hoc Dunn’s test for multiple comparisons was used. For the stress-reactivation experiment, to test whether the observed density of Fos^+^ and tdTom^+^ overlapping VTA cells was higher than chance levels, a two-tailed unpaired *t*-test was used to compare observed overlap density (Fos^+^ and tdTom^+^)/DAPI^+*^100% with chance overlap density (Fos^+^/DAPI^+^)*(tdTom^+^/DAPI^+^)*100%, quantified from the same VTA slices. To test for intrinsic excitability differences between TRAPed and non-TRAPed neurons, One way ANOVAs for biophysical properties, or a Repeated-measures ANOVA for current input - action potential output curves were used. Data in bar graphs are presented as means + SEM. alongside individual data point distributions. Significance level was set to α = 0.05 unless otherwise specified.

## Results

### Acute Social Stress Leads to Fos Expression in a Cellularly Heterogeneous Ventral Tegmental Area Neuronal Ensemble

**FIGURE 1:**
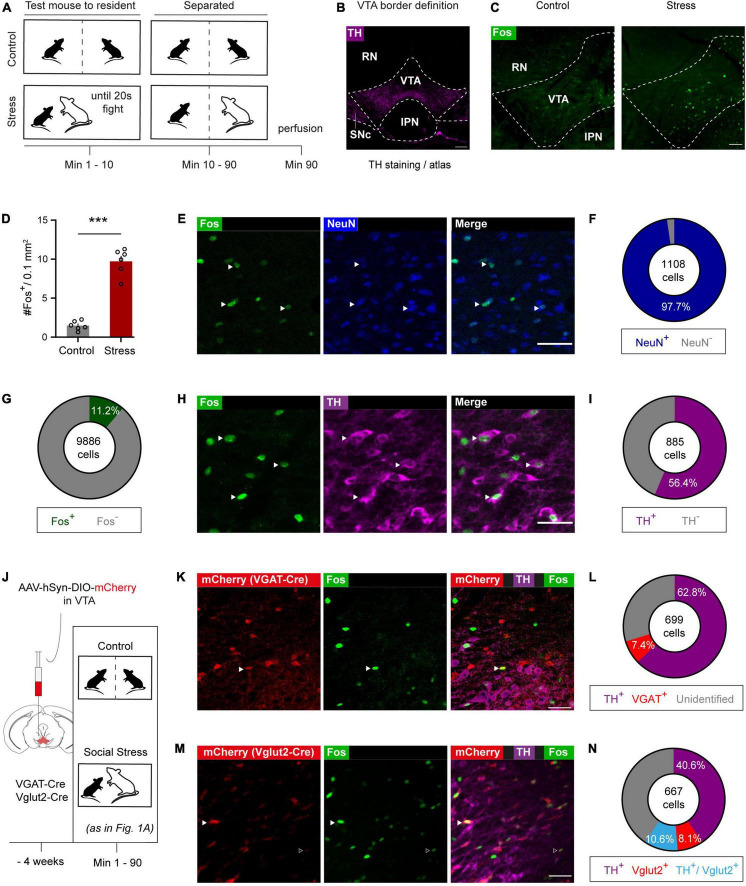
Acute social stress activates a molecularly diverse VTA neuronal ensemble. **(A)** Experimental timeline of acute social stress exposure. **(B)** VTA delineation based on TH staining and Paxinos mouse brain atlas. RN, red nucleus; SNc, substantia nigra compact part; IPN, interpeduncular nucleus. Scale bar: 200 microns. **(C)** Representative images of immunohistochemical staining against Fos in control and socially stressed animals. Dashed lines indicate the VTA borders. Scale bar: 100 microns. **(D)** Bar chart quantification of VTA Fos-expressing cells in control and stress animals. Data are presented as mean + SEM with individual data points (mice) shown alongside of the bars (*n* = 6 per group). **(E)** Representative images of Fos/NeuN co-staining. White arrowheads indicate colocalization of Fos and NeuN. Scale bar: 50 microns. **(F)** Pie chart quantification of extent to which Fos^+^ cells are NeuN^+^ (*n* = 5 mice; pooled data). **(G)** Pie chart quantification of extent to which NeuN^+^ VTA cells are Fos^+^ (*n* = 5 mice; pooled data). **(H)** Representative images of immunohistochemical Fos/TH co-staining. White arrowheads indicate colocalization of Fos and TH. Scale bar: 50 microns. **(I)** Pie chart quantification of extent to which Fos^+^ cells are TH^+^ (*n* = 5 mice; pooled data). **(J)** Viral strategy and timeline used to determine extent of Fos^+^ cells in visualized GABAergic and glutamatergic VTA neurons. **(K)** Representative images of stress-activated (Fos^+^) cells, VGAT (mCherry^+^) and dopaminergic (TH^+^) cells in the VTA of VGAT-Cre mice that underwent acute social stress. White arrowhead indicates colocalization of Fos and VGAT. Scale bar: 50 microns. **(L)** Pie chart characterizing in VGAT-Cre mice which proportion of Fos^+^ cells is TH^+^ and/or mCherry^+^ (*n* = 5 mice; pooled data). **(M)** Representative images of stress-activated (Fos^+^) cells, Vglut2 (mCherry^+^) and dopaminergic (TH^+^) cells in the VTA of Vglut2-Cre mice that underwent acute social stress. Open arrowhead indicates a Fos^+^/Vglut2^+^ neuron. White arrowhead indicates an example triple-labeled Fos^+^/TH^+^/Vglut2^+^ combinatorial neuron. Scale bar: 50 microns. **(N)** Pie chart showing the proportion of dopaminergic, glutamatergic and TH^+^/Vglut2^+^ combinatorial neurons within the stress-activated neuronal ensemble (*n* = 7 mice; pooled data). ***p < 0.001.

To determine the effects of acute social stress on VTA neuronal activation, we briefly exposed mice to an aggressor up until an accumulated total of 20 s of fighting was reached (within 10 min), after which the mice were separated with a semi-permeable barrier (Figure 1A). Afterward we perfused the mice and used expression of the immediate early gene c-Fos as a molecular marker of recent neuronal activity (Morgan and Curran, 1991). We delineated the boundaries of the VTA using the outline of the area immunohistochemically stained for the dopamine marker tyrosine hydroxylase (TH) and using the Paxinos mouse brain atlas (Figure 1B). We found that acute social stress led to an increase of Fos expression in the VTA (Figures 1C,D, *t*_10_ = 11.32; *p* < 0.0001). We further investigated the cellular identity of the Fos^+^ neurons in stressed animals. Co-staining against the neuron-specific marker NeuN confirmed that the vast majority (∼98%) of the Fos^+^ cells were neurons (Figures 1E,F and Supplementary Figure 1A, left). Furthermore, by looking at the number of Fos^+^ cells out of the total NeuN^+^ population, we determined that 11.2% of VTA neurons are activated upon acute social stress (Figure 1G and Supplementary Figure 1A, right). Co-staining against the dopaminergic marker TH revealed that 56.4% of Fos^+^ neurons were dopaminergic (Figures 1H,I and Supplementary Figures 1B,C).

Next, we investigated the presence of Fos in response to stress in VTA GABA and glutamate neurons. To that end we injected vesicular GABA transporter (VGAT)-Cre and vesicular glutamate transporter 2 (Vglut2)-Cre mice in the VTA with an AAV vector leading to Cre-dependent expression of mCherry (Figure 1J and Supplementary Figures 1D,E). Also in these mice, upon acute social stress, we observed a significant increase of Fos^+^ neurons in the VTA (Supplementary Figures 1F,G), with again a major part of these expressing TH (Figures 1K–N and Supplementary Figures 1H–K). In these mice we further established that subsets of VTA_Social stress_ neurons expressed Vglut2 (18.7% of ensemble) or VGAT (7.4%) (Figures 1K–N and Supplementary Figures 1H–K). Finally, within the stress-activated Fos^+^ population, a subset (10.6%) of combinatorial neurons that co-expressed TH and Vglut2 was identified (Figures 1M,N and Supplementary Figure 1K). No neurons within the VTA_Social stress_ ensemble were found to co-express TH and VGAT. Overall these data suggest that acute social stress leads to activation of a substantial VTA neuronal ensemble that is molecularly heterogeneous in terms of neurotransmitter content.

### Social Stress Activates Neurons Present Throughout Ventral Tegmental Area and Its Subregions

Previous studies have suggested the possibility of topographical clustering within the VTA when it comes to encoding aversive signals. An *in vivo* electrophysiology study in rats showed a possible clustering of shock-activated neurons in the ventral VTA (Brischoux et al., 2009), while one in monkeys showed possible clustering of air puff cue activated neurons in the dorsal VTA (Matsumoto and Hikosaka, 2009). Differences in this regard may also exist between medial and lateral VTA cells, as neurons along this axis can have different projection targets and properties (Lammel et al., 2014; Morales and Margolis, 2017).

**FIGURE 2:**
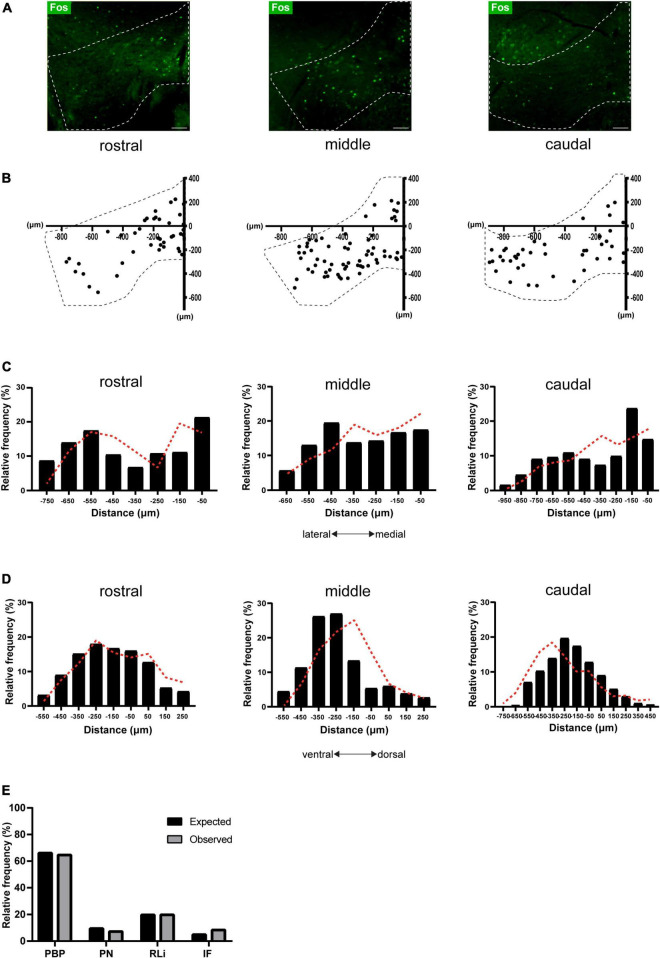
Topographical organization of the VTA_Social stress_ ensemble. **(A)** Representative images of Fos immunostaining in rostral, middle, and caudal VTA slices of stressed animals. Dashed lines indicate the VTA borders. Scale bar: 100 microns. **(B)** 2-D representation of stress-activated cells across the anterior-posterior axis. Dashed lines indicate the VTA borders. Individual points represent anatomically mapped Fos^+^ neurons. Reference point taken at the X–Y axis intersect (0, 0). **(C)** Histogram showing the mediolateral distribution of stress-activated cells, with regards to the midline reference point (see **B**). Black bars represent data obtained from stressed animals (*n* = 7 mice; pooled data; total neurons = 2068, rostral = 569, middle = 787, caudal = 712). Dashed red lines represent the distribution of randomly generated points in a model VTA. **(D)** Histogram showing the Dorsoventral distribution of stress-activated cells, with regards to the midline reference point (see **B**). Black bars represent data obtained from stressed animals (*n* = 7 mice; pooled data; total neurons = 2068, rostral = 569 middle = 787, caudal = 712). Dashed red lines represent the distribution of randomly generated points in a model VTA. **(E)** Proportion of observed (*n* = 7 mice; pooled data) and expected stress-activated cells per VTA subregion: parabrachial pigmented area (PBP), paranigral nucleus (PN), interfascicular nucleus (IF) and the rostral linear nucleus (RLi).

The current experiment aimed to determine if the VTA_Social stress_ ensemble (Figure 1C) has a specific topographical organization. Social stress-driven Fos^+^ expression was clearly visible along the anterior-posterior axis of the VTA (Figure 2A). There was no significant difference in Fos^+^ density between anterior-posterior zones (Supplementary Figure 2A). Next we assessed the topographical organization of the VTA_Social stress_ ensemble along the medial-lateral and the dorsal-ventral axes. To that end, coordinates of all Fos^+^ cells per slice were plotted against a reference point along the midline of the VTA (Figure 2B). We compared the observed topographical distribution of Fos^+^ cells to a distribution of randomly generated points within the VTA geometric boundaries (Supplementary Figure 2B). For the medial-lateral axis, we did not find the observed VTA_Social stress_ ensemble to be different from random in rostral (*z* = –1.301; *p* = 0.097), middle (*z* = 0.268; *p* = 0.606) and caudal (*z* = –1.411; *p* = 0.079) slices (Figure 2C). Similarly, we found no significant differences along the dorsal-ventral axis for rostral (*z* = 0.146; *p* = 0.558), middle (*z* = –1.339; *p* = 0.09) and caudal (*z* = –1.123; *p* = 0.131) VTA slices (Figure 2D).

Another important subdivision of the VTA is in terms of its separate subregions: the PBP, PN, IF and the rostral linear nucleus (RLi) (Lammel et al., 2014; Morales and Margolis, 2017). Therefore we next addressed if the VTA_Social stress_ ensemble was differentially enriched in such VTA subterritories. We used a custom-made (MATLAB) script to automatically map observed Fos^+^ neurons to one of these subregions. Using the same script we now mapped the previously randomly generated points within the geometric boundaries of these VTA subregions (Supplementary Figures 2B,C). The majority of observed Fos^+^ neurons were located in the PBP (∼65%) followed by the RLi (∼20%). Smaller proportions of neurons were located in the IF (∼8%) and PN (∼7%) subregions (Figure 2E). The observed presence of Fos^+^ neurons in different VTA subregions did not differ from their expected presence based on a random topography (Figure 2E, *X*^2^ = 2.84; *df* = 3; *p* = 0.417). Overall these data indicate that the VTA_Social stress_ ensemble is present throughout the VTA, but has no specific topographical clustering.

### TRAP2 Makes the VTA_Social stress_ Ensemble Tractable

**FIGURE 3:**
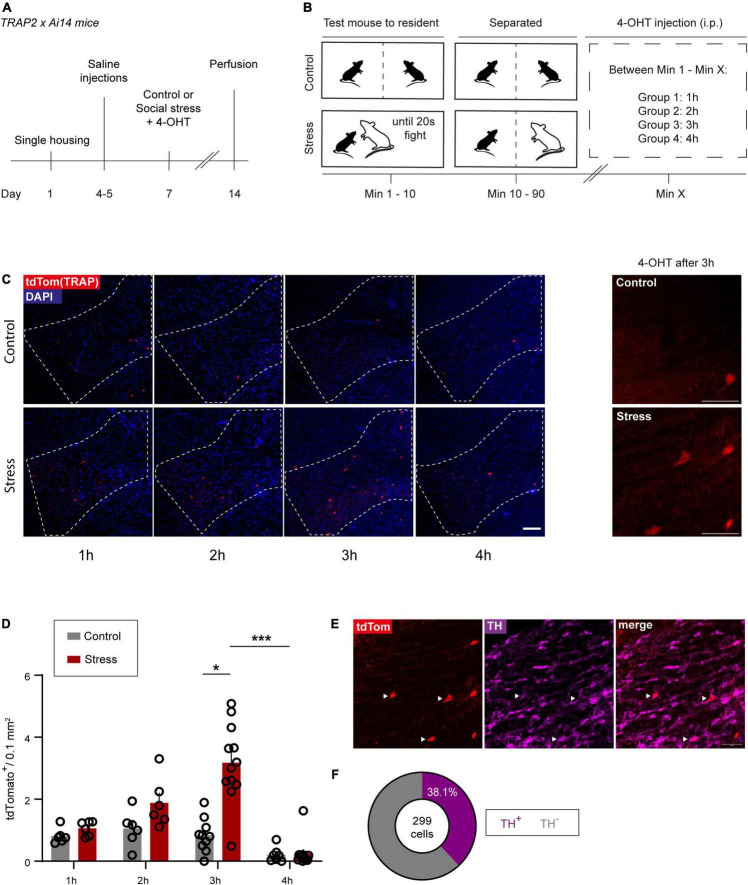
Using TRAP2 to make the VTA_Social stress_ ensemble tractable. **(A)** Experimental timeline. **(B)** Control and social stress paradigms performed on day 7 (see **A**). **(C)** Left: Representative images of VTA sections expressing tdTomato from control and stressed TRAP2xAi14 animals that received 4-OHT at different time points after the start of the experimental session. Scale bar: 100 microns. Right: Higher magnification images of the VTA of control and stress animals that received 4-OHT 3 h after the start of the experimental session. Scale bar: 50 microns. **(D)** Bar chart quantification of VTA control- and stress-TRAPed neurons (1 h: *n* = 6 per group, 2 h: *n* = 6 per group, 3 h_*control*_: *n* = 10, 3 h_*stress*_: *n* = 12, 4 h_*control*_: *n* = 7, 4 h_*stress*_: *n* = 11). Data are presented as mean + SEM with individual data points (mice) shown alongside of the bars. **(E)** Representative images of TH staining and tdTomato expression in a stressed TRAP2xAi14 mouse in the VTA. White arrowheads indicate colocalization of tdTomato (stress-TRAP) and TH. Scale bar: 50 microns. **(F)** Pie chart showing the proportion of dopaminergic neurons within the social stress-TRAPed neuronal ensemble (*n* = 8 mice that received 4-OHT 3 h after stress; pooled data). *p < 0.05; ***p < 0.001.

To gain lasting genetic access to the previously identified VTA_Social stress_ ensemble, we used Targeted Recombination in Active Populations (TRAP2) transgenic mice (DeNardo et al., 2019). This system allows targeted expression of a Cre-dependent effector transgene in neurons that become active during a specific time window. It accomplishes this by using the c-Fos promoter to drive expression of CreER*^T2^* for which the cytosolic transport to the nucleus is tamoxifen-dependent (Supplementary Figure 3A). First we sought to characterize the time window around which TRAPing (i.e., activity-dependent fluorescent labeling) occurs most efficiently. To this end, double transgenic TRAP2xAi14 tdTomato reporter mice were exposed to an accumulated 20 s of fighting social stress after which they remained in the aggressor’s cage separated by a transparent splitter until the time of 4-hydroxytamoxifen (4-OHT) injection (Figures 3A,B). We found a significant increase in the number of stress-TRAPed cells, in comparison to the respective control group, when 4-OHT was injected 3h after the start of the social stress episode (Figures 3C,D, *z* = 3.179; *p* = 0.0413) but not when 4-OHT was injected 1 h (*z* = 0.5457; *p* > 0.99) or 2 h after (*z* = 1.181; *p* > 0.99). Furthermore, when 4-OHT was injected at 4h the number of stress-TRAPed cells was significantly lower than at 3 h (*z* = 5.159; *p* < 0.0001). Finally, little to no VTA neurons (0.04 ± 0.04 cells / 0.01 mm^2^) were TRAPed after social stress in the absence of 4-OHT, confirming the conditionality of the nuclear translocation of Cre (Supplementary Figure 3B).

To further characterize the stress-TRAPed ensemble, we performed immunohistochemistry to label TH, which indicated that 38.1% of the TRAPed neurons were dopaminergic (Figures 3E,F and Supplementary Figure 3C). Notably, this proportion was somewhat lower compared to that of the Fos^+^ identified social stress-sensitive neurons [*t*_(11)_ = 3.567, *p* = 0.0044; Supplementary Figures 1C, 3C – right panel). We next performed topographic analysis on the stress-TRAPed ensemble. We observed that stress-TRAPed cells occurred throughout the anterior-posterior axis of the VTA (Supplementary Figure 3D). Though the density appeared somewhat lower in the caudal zone as compared to the middle and rostral zone, this difference did not survive multiple comparison testing [One Way ANOVA, *F*_(2,15)_ = 4.276, *p* < 0.05; Bonferroni corrected *post hoc*: Caudal vs. Rostral *t*_15_ = 2.412, *p* = 0.087; Middle vs. Caudal *t*_15_ = 2.638, *p* = 0.056; Rostral vs. Middle *t*_15_ = 0.2261, *p >* 0.999]. To further assess the topography we evaluated whether the stress-TRAPed ensemble had specific clustering within VTA subregions. We observed that most of the stress-TRAPed cells were in the PBP (58.3%; Supplementary Figure 3E). The density of stress-TRAPed cells across subregions was similar to that of a randomly generated distribution (Supplementary Figure 3E; *X*^2^ = 4.57; *df* = 3; *p* = 0.2059). Furthermore, we found that the topographic distribution of VTA stress-TRAPed cells was similar to that of VTA_Social stress_ neurons identified instead with Fos immunohistochemistry [Two-way ANOVA, Interaction Subregion × Labeling approach, *F*_(3,44)_ = 2.072, *p* = 0.1176, *ns*; Supplementary Figure 3F]. Despite similar topography between the Fos immunohistochemically defined VTA_Social stress_ ensemble and the stress-TRAPed ensemble (4-OHT after 3 h), it is notable that the number of cells in the latter was significantly lower (∼3 fold) (Figures 1D, 3D; *t*_16_ = 9.33; *p* < 0.0001).

Acutely encountering a stressor that is new can represent a different experience from encountering that stressor when it is more familiar, and this may engage partially distinct neuronal ensembles (Tanaka et al., 2012; Bains et al., 2015; Ye et al., 2016). We next used the TRAP2 approach to determine the extent to which the stress-TRAPed ensemble (responding to the first social stress exposure) is also reactivated upon exposure to a second social stressor. To that end we exposed a subset of TRAP2xAi14 mice first to social stress, combined with 4-OHT injection 3 h later. After 3 weeks we re-exposed these mice to social stress (now in the absence of a 4-OHT injection, thus not further TRAPing neurons; Supplementary Figure 3B) and perfused the animals 90 min later to compare immunohistochemical Fos responses to the second social stressor, with TRAPing by the first social stressor (Supplementary Figure 3G). We found that of all VTA neurons that were responsive to the 2nd stressor (i.e., Fos^+^) 11.9% had been stress-TRAPed upon exposure to the first stressor. Inversely, 49.4% of the stress-TRAPed neurons (i.e., neurons responsive to the first stressor) were again responsive to the second stressor (i.e., Fos^+^) (Supplementary Figures 3H,I). We assessed whether reactivity to the second stressor occurred to a higher degree within the stress-TRAPed ensemble. To that end we contrasted the percentage of VTA neurons responsive to the second stressor within the (first) stress-TRAPed VTA population (Fos^+^/tdTom^+^) to that within the entire VTA cellular population (Fos^+^/DAPI^+^). We found that there was a considerable overrepresentation of cells responsive to the second social stressor amidst the first social stress-TRAPed cells [Supplementary Figure 3J; *t*_(10)_ = 13.31, *p* < 0.0001]. Moreover, we checked the extent to which the reactivation in the TRAPed ensemble was higher than what would be expected to occur by chance due to the density of Fos and the density of tdTomato labeling in the VTA. We calculated the density of observed overlap of tdtomato^+^ and Fos^+^ cells amidst all VTA cells [i.e., (Fos^+^ and tdTom^+^)/DAPI^+^ * 100%)], and contrasted it with its expected overlap due to chance [i.e., (Fos^+^/DAPI^+^) * (tdTom^+^/DAPI^+^) * 100%]. We found that the observed degree of overlap was clearly higher than expected overlap at chance levels [Supplementary Figure 3K; *t*_(10)_ = 4.736, *p* = 0.0008].

Overall, here we provide insight in the time window around which a VTA_Social stress_ neuronal ensemble can be optimally captured using TRAP2. We also show that a social stress-TRAPed ensemble, while having a few differences with a Fos immunohistochemistry-identified ensemble, importantly shares key features with such an ensemble.

### The Stress-Activated Ventral Tegmental Area Ensemble Shows Higher Excitability Than Its Neighbor Cells

**FIGURE 4:**
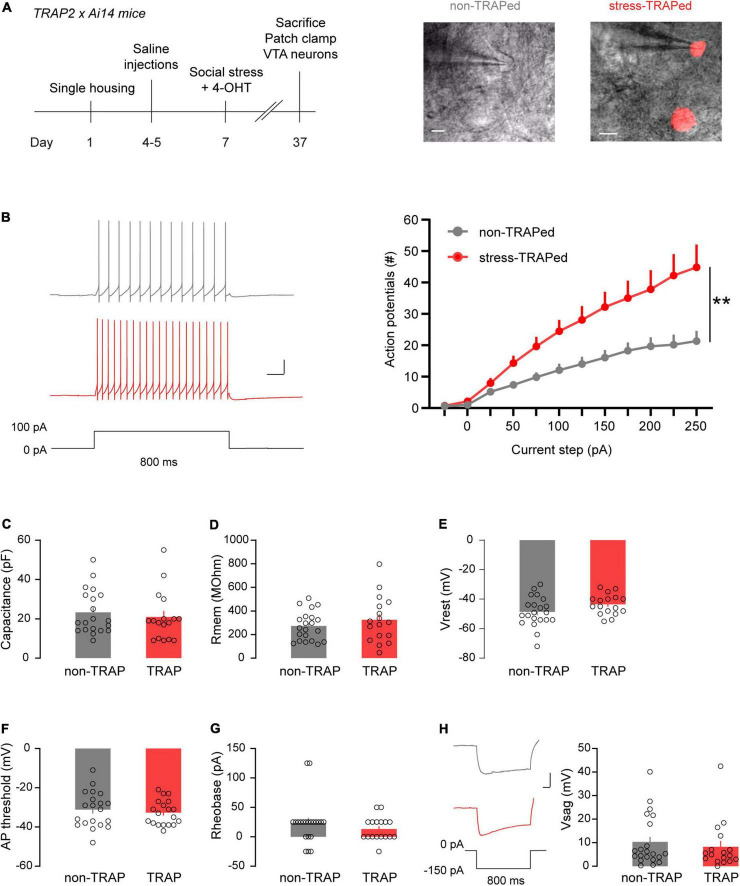
Electrophysiological characterization of the VTA_Social stress_ ensemble. **(A)** Left: Timeline of patch clamp experiment of stress-TRAPed and non-TRAPed VTA neurons. Right: Examples of patch clamped non-TRAPed and stress-TRAPed (red) VTA neurons (Scale bars: 50 microns). **(B)** Left: Representative traces showing firing of a non-TRAPed (gray) and stress-TRAPed (red) neuron during a 800 ms current step of 100 pA (Scale bars: 20 mV, 100 ms). Right: Line plot showing the number of action potentials fired by VTA neurons as a function of injected current steps (non-TRAPed *n* = 21 cells, stress-TRAPed *n* = 17 cells, *n* = 9 mice). Data are presented as mean + SEM. **(C)** Bar chart showing the capacitance of non-TRAPed and stress-TRAPed neurons. Data are presented as mean + SEM, with individual data points (cells) plotted alongside (non-TRAPed *n* = 21 cells, stress-TRAPed *n* = 17 cells, *n* = 9 mice). **(D)** Bar chart showing the membrane resistance (Rmem) of non-TRAPed and stress-TRAPed neurons. Data are presented as mean + SEM, with individual data points (cells) plotted alongside (non-TRAPed *n* = 21 cells, stress-TRAPed *n* = 17 cells, *n* = 9 mice). **(E)** Bar chart showing the resting membrane potential (Vrest) of non-TRAPed and stress-TRAPed neurons. Data are presented as mean + SEM with individual data points (cells) plotted alongside (non-TRAPed *n* = 21 cells, stress-TRAPed *n* = 17 cells, *n* = 9 mice). **(F)** Bar chart showing the action potential (AP) threshold of non-TRAPed and stress-TRAPed neurons. Data are presented as mean + SEM, with individual data points (cells) plotted alongside (non-TRAPed *n* = 20 cells, stress-TRAPed *n* = 16 cells, *n* = 9 mice). **(G)** Bar chart showing the rheobase of non-TRAPed and stress-TRAPed neurons. Data are presented as mean + SEM, with individual data points (cells) plotted alongside (non-TRAPed *n* = 21 cells, stress-TRAPed *n* = 17 cells, *n* = 9 mice). **(H)** Left: Example of voltage sags (Vsag) in response to –150 pA of injected current for 800 ms in non-TRAPed (gray) and stress-TRAPed (red) neurons. Scale bars: 20 mV, 100 ms. Right: Bar chart showing the Vsag of non-TRAPed and stress-TRAPed neurons. Data are presented as mean + SEM, with individual data points (cells) plotted alongside (non-TRAPed *n* = 21 cells, stress-TRAPed *n* = 17 cells, *n* = 9 mice). **p < 0.01.

We next sought to determine whether the social stress ensemble of VTA neurons differs compared to cells not part of this ensemble in terms of electrophysiological properties. To this end TRAP2xAi14 mice were injected with 4-OHT 3 h after they were subjected to an episode of acute social stress (Figure 4A). Afterward, animals were housed alone for a minimum of 4 weeks before electrophysiological recordings were done. This relatively long waiting period was chosen to attempt to primarily capture basal features of the ensemble rather than the (acute) effects of a prior stressor on it. We patch clamped both VTA neurons activated by social stress (i.e., red fluorescent) and their non stress-activated neighboring neurons. These neurons are hereafter termed stress-TRAPed vs. non-TRAPed neurons, respectively (Figure 4A). We recorded these neurons in whole cell current clamp mode and evaluated their intrinsic properties. Stress-TRAPed neurons were more excitable (i.e., fired more action potentials) when injecting positive current steps, compared to neighboring non-TRAPed neurons [Figure 4B; Repeated Measures ANOVA: main effect TRAP *F*_(1,34)_ = 8.42, *p* < 0.01].

Since the VTA_Social stress_ ensemble comprises multiple cell types, it is possible that the observed differences in excitability occurred due to biased sampling from dopaminergic and non-dopaminergic neurons in the stress-TRAPed and non-TRAPed populations. To address this possibility we labeled a subset of patched neurons with biocytin to identify whether these neurons were dopaminergic by staining for TH, after the recording (Supplementary Figure 4A). In this subset, 50% of non-TRAPed and 44% of stress-TRAPed neurons were dopaminergic (Supplementary Figure 4B). We observed that the higher excitability in the stress-TRAPed population occurred independently of whether or not cells expressed TH [Repeated Measures ANOVA, Main effect TRAP, *F*_(1,17)_ = 6.96, *p* < 0.05; Main effect TH: *F*_(1,17)_ = 2.29, *p* = 0.15*;* Interaction TRAP × TH: *F*_(1,17)_ = 0.001, *p* = 0.97; Supplementary Figure 4C]. Notable differences in VTA cellular electrophysiological properties and firing patterns have particularly been described to occur across the medial-lateral axis of the VTA (Lammel et al., 2014). To ensure that the different electrophysiological phenotype was not due to accidental oversampling of stress-TRAPed cells in one segment over the other, we also compared the excitability of stress-TRAPed and non-TRAPed neurons in medial versus lateral VTA. We found that both in the medial VTA and in the lateral VTA there was increased excitability in VTA stress-TRAPed neurons compared to their neighbors [Supplementary Figure 4D, Repeated Measures ANOVA, Main effect TRAP *F*_(1,34)_ = 8.41, *p* = 0.006; Main effect location *F*_(1,34)_ = 0.17, *p* = 0.68; Interaction TRAP*location *F*_(1,34)_ = 0.06, *p* = 0.81].

We examined whether there were other biophysical parameters that differed between stress-TRAPed and non-TRAPed neurons. We did not observe differences between these cellular populations in terms of their electrical capacitance, a proxy of cell size [*F*_(1,36)_ = 0.21, ns; Figure 4C]. The two populations also did not differ in terms of membrane resistance [*F*_(1,36)_ = 1.02, ns; Figure 4D], which via Ohmic properties determines the voltage deflection obtained by a given injected current. The populations also did not differ in terms of their resting membrane potential [*F*_(1,36)_ = 3.02, ns; Figure 4E], action potential threshold [*F*_(1,34)_ = 0.11, ns; Figure 4F], or the minimal input current required to elicit the first action potential [i.e., the rheobase, *F*_(1,936)_ = 1.06, ns; Figure 4G]. Finally, the populations did not differ in the magnitude of their voltage sag, a hyperpolarization-induced depolarization mediated by H-currents (Friedman et al., 2017), in response to a negative current injection of –150 pA [*F*_(1,36)_ = 0,36, ns; Figure 4H].

Overall these data suggest that VTA stress-TRAPed neurons are more excitable than non-TRAPed neighboring VTA neurons. This difference was not due to biased sampling from distinct VTA populations in terms of neurotransmitter content, nor due to biased sampling from topographically distinct regions in the VTA. This suggests that both dopaminergic and non-dopaminergic VTA_Social stress_ neurons across the VTA, exhibit higher excitability than their neighbors. Notably, aside from differences in the maximal amount of action potentials that VTA_Social stress_ neurons could fire, they did not otherwise present with a different biophysical profile from their neighbor cells.

## Discussion

We used Fos mapping and TRAP2 transgenic mice to identify and characterize a social stress-responsive neuronal ensemble in the VTA. We find these cells to be a specific set of neurons with higher excitability. We also demonstrate how the TRAP2 approach can be used to make this VTA_Social stress_ ensemble controllable for targeted investigations of their properties and function. Below we further discuss these findings.

### Neurotransmitter Identity of the Ventral Tegmental Area Social Stress Ensemble

We found that a substantial portion of the stress-responsive VTA neuronal ensemble is TH-positive. This is in accordance with other work showing that at least subsets of VTA dopaminergic neurons are acutely excited by aversive stimuli (Brischoux et al., 2009; Matsumoto and Hikosaka, 2009; Cohen et al., 2012; Cai et al., 2020). We also found a portion of glutamate neurons (i.e., Vglut2-positive) and a small portion of GABA neurons (i.e., VGAT-positive) as part of the ensemble. This is in accordance with previous work showing sensitivity of VTA GABA and glutamate neurons to aversive signals other than social stress. For instance, within the population of VTA GABA neurons there are at least subsets excited by electric shocks (Tan et al., 2012; Root et al., 2020), restraint stress (Sun et al., 2021), and air puffs (Cohen et al., 2012). Within the population of VTA glutamate neurons there are at least subsets that are stimulated by looming threats (Barbano et al., 2020), threatening odors (Barbano et al., 2020), and electric shocks (Root et al., 2020). Experimental manipulations of these neuronal populations can also drive behaviors in accordance with adaptions to stressful situations. Optogenetic stimulation of VTA GABA neurons can drive conditioned place avoidance (Tan et al., 2012) and disrupts reward consumption (Van Zessen et al., 2012). Ablation of VTA glutamate (but not VTA GABA) neurons, or their chemogenetic silencing, reduces innate escape behaviors to threatening stimuli such as looming shadows or predator odors (Barbano et al., 2020). These studies show that at least part of the total populations of VTA GABA and glutamate neurons can participate in the detection of aversive signals and the shaping of subsequent adaptive behaviors.

The VTA also contains neurons capable of coreleasing combinations of dopamine, glutamate and GABA (Morales and Margolis, 2017). Regarding VTA neurons coreleasing dopamine-glutamate, we found a substantial subpopulation of both TH-positive and Vglut2-positive neurons amongst the VTA_Social stress_ ensemble. VTA neurons expressing TH and Vglut2 have been previously described to be medially positioned in the VTA (Morales and Margolis, 2017; Eskenazi et al., 2021). Their behavioral functions have thus far been mainly studied using (conditional) removal of Vglut2 from dopaminergic neuronal populations, to thereby attenuate the corelease. Though some studies suggest that this can alter proclivity for anxiety, other studies do not confirm this (Eskenazi et al., 2021). The role that these neurons may have in stress-driven behaviors therefore remains to be further elucidated. Regarding VTA neurons coreleasing dopamine-GABA: although we did not find combinatorial cells that were both TH-positive and VGAT-positive within the VTA_Social stress_ ensemble, it should be noted that GABA co-release from dopamine midbrain neurons has mainly been described to occur via non-canonical mechanisms (Tritsch and Sabatini, 2012; Berrios et al., 2016; Morales and Margolis, 2017). Indeed the vesicular packaging mechanisms underlying GABA corelease in subsets of midbrain dopamine neurons may primarily involve the vesicular monoamine transporter 2 (VMAT2), which also accrues dopamine in vesicles (Tritsch and Sabatini, 2012; Berrios et al., 2016). Therefore, the observed absence of a TH-positive/VGAT-positive population within the VTA_Social stress_ ensemble does not rule out the possibility that some of the TH-positive neurons in the ensemble do functionally corelease GABA. Finally, regarding VTA neurons coreleasing glutamate-GABA, the current study did not allow for the evaluation of the presence of both VGAT-positive and Vglut2-positive combinatorial neurons. This is because here we opted to identify VTA GABAergic and glutamatergic neurons using transgenic Cre lines combined with virally driven Cre-dependent expression of fluorophores. This approach has the key advantage of giving much better signal to noise ratio when identifying GABAergic and glutamatergic somata in the VTA than is obtainable with immunohistochemical techniques, allowing for more confident and accurate colocalization assessments (Taylor et al., 2014). The disadvantage is that in this study we did not study potential VGAT-positive and Vglut2-positive neurons in the stress ensemble. These combinatorial neurons can instead be studied using intersectional transgenic approaches (e.g., using both Cre and FLP lines). Using such intersectionality, VTA combinatorial neurons positive for both Vglut2 and VGAT have been recently shown to be excited by not only rewards but also by electric shocks (Root et al., 2020). While it is possible that some of the VGAT-positive neurons in the VTA_Social stress_ ensemble mapped in our study do also express Vglut2, their relatively low presence in the ensemble to begin with (∼7%), suggests that at most this combination would constitute a minority in the ensemble.

In this study we compared the social stress-activated ensemble in the VTA, with another relevant experience as a control (i.e., a novel social experience with a new cage mate). Novelty can engage the VTA (Bromberg-Martin et al., 2010), but did so considerably less than the social stress (also with a novel mouse) experience. Interestingly, in the C57Bl6J Fos immunohistochemistry experiments and in the TRAP experiments we found that the control ensemble was not only smaller, but also that its molecular composition contained a lower proportion of TH^+^ cells than the social stress ensemble. Of note is that this was different in the VGAT-Cre and Vglut2-Cre mice. In those mice, the control Fos^+^ ensemble (i.e., the effect of the novel cage mate) was somewhat larger in density (∼5 Fos+ cells/0.1 mm2 vs ∼1.5 Fos+ cells/0.1 mm2) than was the case in C57Bl6J mice and TRAPxAi14 mice. This could reflect differences across mouse lines in the extent to which a novel cage mate experience engages the VTA. Alternatively, it may be that the prior (partially stressful) stereotactic surgery that VGAT-Cre and Vglut2-Cre, but not the C57Bl6J or TRAPxAi14, mice underwent for labeling purposes impacted on the processing of such a subsequent stimulus.

Overall, in this study we describe that a significant portion of VTA_Social stress_ neurons are dopaminergic, with smaller subsets comprising GABAergic, glutamatergic and combinatorial dopaminergic-glutamatergic neurons.

### TRAPing a Ventral Tegmental Area Social Stress Ensemble

Here we validated the use of TRAP2 in the context of social stress and gained lasting genetic access to implicated VTA neurons. We found that injecting 4-OHT 3h after the start of the stress episode resulted in optimal (in terms of number of labeled cells) TRAPing of VTA cells. Notably, we used an aqueous vehicle, which leads to rapid and transient presence of 4-OHT in the mouse brain (peaking around 1h after systemic injection) (Ye et al., 2016). This allows for a more narrow time window to TRAP neurons that are active during an experience than with an oil-based vehicle (Ye et al., 2016; DeNardo et al., 2019). Our data suggest that at least for VTA cells, giving aqueous 4-OHT 3 h after the start of the social stress experience results in strong temporal convergence of c-Fos promotor-driven cytosolic CreER*^T2^* expression and of the cellular 4-OHT that chaperones CreER^T2^ to the nucleus for subsequent fluorophore recombination (Supplementary Figure 3A). A longer separation between social stress and 4-OHT administration may mean cytosolic CreER^T2^ levels have become too low for efficient recombination. Instead, a shorter separation may have caused VTA cellular 4-OHT levels to prematurely peak with regards to development of optimal CreER^T2^ cytosolic presence. Overall, our study gives practical insight in how to TRAP a VTA_Social stress_ ensemble.

How does this TRAPed VTA_Social stress_ ensemble compare to the ensemble observed with Fos immunohistochemistry? One difference is the amount of cells implicated with both approaches. The TRAP2xAi14 approach with the optimal 3 h time window yielded fewer social stress-reactive neurons (∼3-fold difference) than did the Fos immunohistochemistry approach. While this indicates that the TRAP2 approach does not capture all the Fos protein expressing neurons in the VTA, several lines of evidence suggest that it does capture not only a considerable, but also a representative portion of it. First, both with Fos immunohistochemistry and the TRAP2 approach we found that a significant portion of neurons were TH-positive (albeit somewhat lower with the TRAP approach), and had similar topographical positioning throughout the VTA. Moreover, both in C57Bl6J mice using Fos immunohistochemistry and in TRAP2xAi14 mice we observed that under control conditions (i.e., exposure to a novel conspecific) we obtained an ensemble in which TH presence was also comparable (∼20%). Furthermore, even though the first experience of an acute exposure to a (social) stressor can be physiologically different from the experience of a subsequent exposure (Tanaka et al., 2012; Bains et al., 2015), we did find that a large proportion of stress-TRAPed cells (considerably higher than in non-TRAPed cells) was reactivated by a second social stressor. The extent of this reactivation is also in line with reactivation extents in previous studies using TRAP approaches in the context of fear memory processes and foot shocks (Ye et al., 2016; DeNardo et al., 2019).

A recent study using a previous version of the TRAP approach (TRAP1) found that restraint stress activated a population of VTA neurons. Interestingly, this ensemble was partially dopaminergic, but mostly GABAergic (Sun et al., 2021). There are notable differences between TRAP1 and TRAP2 as an approach, with the latter being less disruptive for endogenous Fos levels and having a more efficient form of Cre (Guenthner et al., 2013; Allen et al., 2017; DeNardo et al., 2019). Potential methodological differences notwithstanding, it is an interesting possibility that different stressors engage partially different VTA ensembles. Indeed previous studies have shown that different stressors (including social stress versus restraint stress) can engage different ensembles within regions such as the claustrum and the hypothalamus (Nakamura et al., 2022; Niu et al., 2022). The extent to which this occurs in the VTA is an interesting line of future research.

Overall, our study demonstrates how to TRAP a VTA_Social stress_ ensemble that is in many aspects representative of one identified instead through Fos immunohistochemistry. This permits the use of this approach to address functional properties of these VTA_*Social stress*_ cells.

### Electrophysiological Properties of a TRAPed VTA_Social stress_ Ensemble

Capitalizing on the usefulness of TRAP2 approaches to identify VTA_Social stress_ neurons in non-fixed tissue, we used *ex vivo* patch-clamp electrophysiology to characterize their properties. Compared to their neighboring cells, VTA_Social stress_ neurons fired more action potentials when injected with increasing amounts of current. The contrast with neighboring cells suggests that this was not due to biased sampling from different VTA regions. Further in accordance with this, both in the medial and in the lateral VTA, a relevant axis when it comes to neurophysiological signatures (Lammel et al., 2014), we observed that VTA_Social stress_ cells were able to fire at higher frequencies. This difference was also unlikely due to different molecular cell types sampled from, as it was also present in a subset of biocytin-labeled cells with around half of the neurons being dopaminergic for both the VTA_Social stress_ cells and for their neighbors. Overall this suggests that both dopaminergic and non-dopaminergic VTA_Social stress_ cells are electrophysiologically distinct from their neighbors.

There is a possibility that the enhanced excitability of the VTA_Social stress_ neurons is not due to a basal feature of the ensemble *per se*, but represents a specific and very protracted adaptation of just these cells (but not their neighbor cells) to the much earlier acute social stress experience. While this cannot be ruled out, we consider it unlikely as there were 4 weeks between the initial TRAP event (i.e., a singular social stress episode) and the subsequent recording. Previous work has shown that with chronic (rather than acute) social stress VTA dopamine neurons can become hyperexcitable via a mechanism involving increased hyperpolarization-activated current (H-current) (Friedman et al., 2017). Instead, in our VTA_Social stress_ neurons the hyperexcitability occurred in the absence of differences in the voltage sag (a voltage read-out of the H-current) between these cells and their neighbors.

The mechanisms underlying the heightened excitability of the VTA_Social stress_ neurons remains unclear. Aside from the aforementioned absence of a difference in voltage sag, these neurons did not differ from their neighbors in other biophysical properties such as action potential threshold, resting membrane potential, or membrane resistance. The observed difference between these cells in terms of how many action potentials they can fire in response to strong input could have various mechanistic origins. One possibility is that a difference exists in the balance of excitatory and inhibitory synaptic inputs impinging on both types of VTA cells, which differentially contributes to the action potentials they are able to fire. Notably, however, the difference was mainly present at higher levels of direct current injection into the cells, suggesting involvement of intrinsic mechanisms. A more likely possibility is then that distinct intrinsic properties other than the aforementioned unaltered ones cause the difference. This may include specific membrane ion channel compositions of VTA_Social stress_ cells, compared to their neighbors, which permit them to have less spike adaptation to strong excitatory input. Future studies will need to address the mechanistic basis of VTA_Social stress_ hyperexcitability.

Overall this study characterizes an ensemble of neurons in the VTA that is directly sensitive to social stress. It demonstrates that neurons of different molecular identities are part of the ensemble, and that the ensemble is present throughout the VTA. The study also provides insight in how to use TRAP2 approaches to make this population tractable, and presents evidence that this population has a distinctive capability of high frequency firing. These insights will facilitate future studies in addressing how naturalistic social stressors alter reward circuitry, which is relevant in the context of various pathologies, including obesity, drug addiction and depression (Chaudhury et al., 2013; Friedman et al., 2014; Razzoli et al., 2015; Holly and Miczek, 2016; Newman et al., 2018).

## Data Availability Statement

The raw data supporting the conclusions of this article will be made available by the authors upon request, without undue reservation.

## Ethics Statement

The use of experimental animals in this study was reviewed and approved by the Dutch National Central Authority for Scientific Procedures on Animals (CCD).

## Author Contributions

IK performed and analyzed the immunohistochemical staining and TRAP2 experiments. IK and SS analyzed the topographical distributions of Fos^+^ and stress-TRAPed neurons. LL acquired and analyzed the brain slice electrophysiological data. IK and IW-D performed the stereotactic surgeries. FM and IK designed the study and wrote the manuscript with the help of all authors. All authors contributed to the article and approved the submitted version.

## Conflict of Interest

The authors declare that the research was conducted in the absence of any commercial or financial relationships that could be construed as a potential conflict of interest.

## Publisher’s Note

All claims expressed in this article are solely those of the authors and do not necessarily represent those of their affiliated organizations, or those of the publisher, the editors and the reviewers. Any product that may be evaluated in this article, or claim that may be made by its manufacturer, is not guaranteed or endorsed by the publisher.

## Footnotes

1
https://fiji.sc

## Supplementary Material

Supplementary Figure 1 **(A)** Left: Bar chart showing the proportion of NeuN^+^ cells within the subset of stress-implicated Fos^+^ cells across animals (*n* = 5 mice). Right: Bar chart showing the proportion of Fos^+^ cells out of all NeuN^+^ VTA cells across animals (*n* = 5 mice). **(B)** Pie chart showing the proportion of dopaminergic neurons in control-activated VTA neurons (*n* = 5 mice). **(C)** Bar chart showing the proportion of TH^+^ cells out of all Fos^+^ cells across animals (*n*_*control*_ = 5 mice, *n*_*stress*_ = 5 mice). **(D)** Representative image showing distribution of GABAergic neurons in the VTA, after viral delivery of AAV-hSyn-DIO-mCherry in VGAT-Cre mice. Scale bar: 200 microns. **(E)** Representative image showing distribution of glutamatergic neurons in the VTA, after viral delivery of AAV-hSyn-DIO-mCherry in Vglut2-Cre mice. Scale bar: 200 microns. **(F)** Bar chart showing the quantification of VTA Fos^+^ neurons in VGAT-Cre control and stressed animals (*n*_*control*_ = 6, *n*_*stress*_ = 5 mice). Data are presented as mean + SEM, with individual data points (cells) plotted alongside. **(G)** Bar chart showing the quantification of VTA Fos^+^ neurons in Vglut2-Cre control and stressed animals (*n*_*control*_ = 7, n_*stress*_ = 7 mice). Data are presented as mean + SEM, with individual data points (cells) plotted alongside. **(H)** Pie chart showing the molecular identity of control-activated VTA neurons in VGAT-Cre animals (*n* = 6 mice; pooled data). **(I)** Bar chart showing the molecular identity of Fos^+^ cells in VGAT-Cre animals across animals (*n*_*control*_ = 6, *n*_*stress*_ = 5 mice). **(J)** Pie chart showing the molecular identity of control-activated VTA neurons in Vglut2-Cre animals (*n* = 7 mice; pooled data). **(K)** Bar chart showing the molecular identity of Fos^+^ cells in Vglut2-Cre animals (*n*_*control*_ = 7, *n*_*stress*_ = 7). ***p* < 0.01.

Supplementary Figure 2 **(A)** Bar chart showing the number of Fos^+^ neurons in different zones along the anterior-posterior axis of the VTA: rostral, middle and caudal. **(B)** Randomly generated (simulated) points (red) inside the geometric boundaries of the VTA (light blue area). Number of points was equal to the total number of experimentally observed Fos^+^ cells in the respective AP zone (*n*_*rostral*_ = 569; *n*_*middle*_ = 787; *n*_*caudal*_ = 712). **(C)** Model VTA subregion delineation, based on Paxinos mouse brain atlas. Parabrachial pigmented area (PBP; beige), paranigral nucleus (PN; violet), interfascicular nucleus (IF; yellow) and the rostral linear nucleus (RLi; blue).

Supplementary Figure 3 **(A)** Schematic depiction of TRAP2 mechanism of action. Upon social stress, c-Fos promoter drives the expression of Fos and iCreER^T2^ (tamoxifen-dependent Cre). In the presence of 4-OHT, Cre translocates to the cell nucleus where it induces recombination of the fluorescent reporter tdTomato. **(B)** Left: Representative image of a VTA section of an animal that underwent social stress in the absence of 4-OHT injection. Scale bar: 200 microns. Right: Bar chart quantification of stress-TRAPed VTA cells in the absence of 4-OHT (*n* = 2 mice). **(C)** Left: Pie chart showing the proportion of dopaminergic cells within a control-TRAPed VTA neuronal ensemble (*n* = 6 TRAP2xAi14 mice that received 4-OHT 3 h after the start of a control session; pooled data). Right: Bar chart showing the proportion of TH^+^ cells out of TRAPed cells across animals (*n*_*control*_ = 6 mice, *n*_*stress*_ = 8 mice). **(D)** Bar chart showing the number of stress-TRAPed neurons in different zones along the anterior-posterior axis of the VTA: rostral, middle and caudal across animals (*n* = 6 mice). **(E)** Proportion of observed and expected stress-TRAPed cells per VTA subregion: parabrachial pigmented area (PBP), paranigral nucleus (PN), interfascicular nucleus (IF) and the rostral linear nucleus (RLi). **(F)** Bar chart showing the VTA subregional distribution of acute stress implicated Fos^+^ and stress-TRAPed cells across animals [*n*_*Fos*_ = 7 mice, *n*_*tdTom(stress–TRAP)*_ = 6 mice]. **(G)** Experimental timeline to determine the extent to which the stress-TRAPed neuronal ensemble is reactivated by a second social stressor. **(H)** Representative images of Fos staining in VTA slices of a mouse that underwent stress-TRAPing 3 weeks earlier. White arrowheads indicate colocalization of Fos and tdTomato. Scale bar: 50 microns. **(I)** Left: Pie chart showing the proportion of stress-reactivated neurons (#double Fos^+^ and TRAPed out of total TRAPed). Right: Pie chart showing the proportion of TRAPed neurons out of the total Fos^+^ population (#double TRAPed and Fos^+^ out of total Fos^+^) *n* = 6 mice (pooled data). **(J)** Bar chart showing the frequency of social stress reactivated neurons within the stress-TRAPed ensemble in the VTA. Left: Percentage of Fos^+^ cells out of all VTA cells after the second stress episode (DAPI^+^; *n* = 6 mice). Right: Percentage of Fos^+^ cells in response to the second stressor within the population of VTA cells that were TRAPed during the first stress episode (tdTom^+^; *n* = 6 mice). **(K)** Bar chart showing the observed density of double labeled (Fos^+^ and tdTom^+^) cells within the VTA, as compared to the chance expected density of double labeled cells (*n* = 6 mice). ****p* < 0.001; *****p* < 0.0001.

Supplementary Figure 4 **(A)** Representative image of fluorescently labeled biocytin filled neurons (green), stained for dopamine marker TH (red). White arrowhead indicates a neuron positive for biocytin and TH. Scalebar: 10 microns. **(B)** Pie charts of the amount of TH^+^ and TH^–^ cells within the biocytin filled cellular population in non-TRAPed (*n* = 12 cells) and stress-TRAPed neurons (*n* = 9 cells). **(C)** Left: Line plot showing the number of action potentials fired as a function of injected current in all biocytin-labeled VTA neurons in non-TRAPed cells (*n* = 12 cells) versus stress-TRAPed cells (*n* = 9 cells). Right: Line plot showing the separate curves for TH^+^ non-TRAPed (*n* = 6) vs. stress-TRAPed (*n* = 4), and TH^–^ non-TRAPed (*n* = 6 cells) vs. stress-TRAPed (*n* = 5 cells). **(D)** Left: Line plot showing the number of action potentials fired by non-TRAPed (*n* = 10 cells) and stress-TRAPed cells (*n* = 8 cells) in the lateral VTA. Right: Line plot showing the number of action potentials fired by non-TRAPed (*n* = 11 cells) and stress-TRAPed cells (*n* = 9 cells) in the medial VTA. **p* < 0.05, ***p* < 0.01.
