# Supplementary figures and images for: Characterizing and TRAPing a Social Stress-Activated Neuronal Ensemble in the Ventral Tegmental Area
Source: Front Behav Neurosci. 2022 Jul 8;16:936087. doi: 10.3389/fnbeh.2022.936087 (PMC9304991; doi:10.3389/fnbeh.2022.936087)

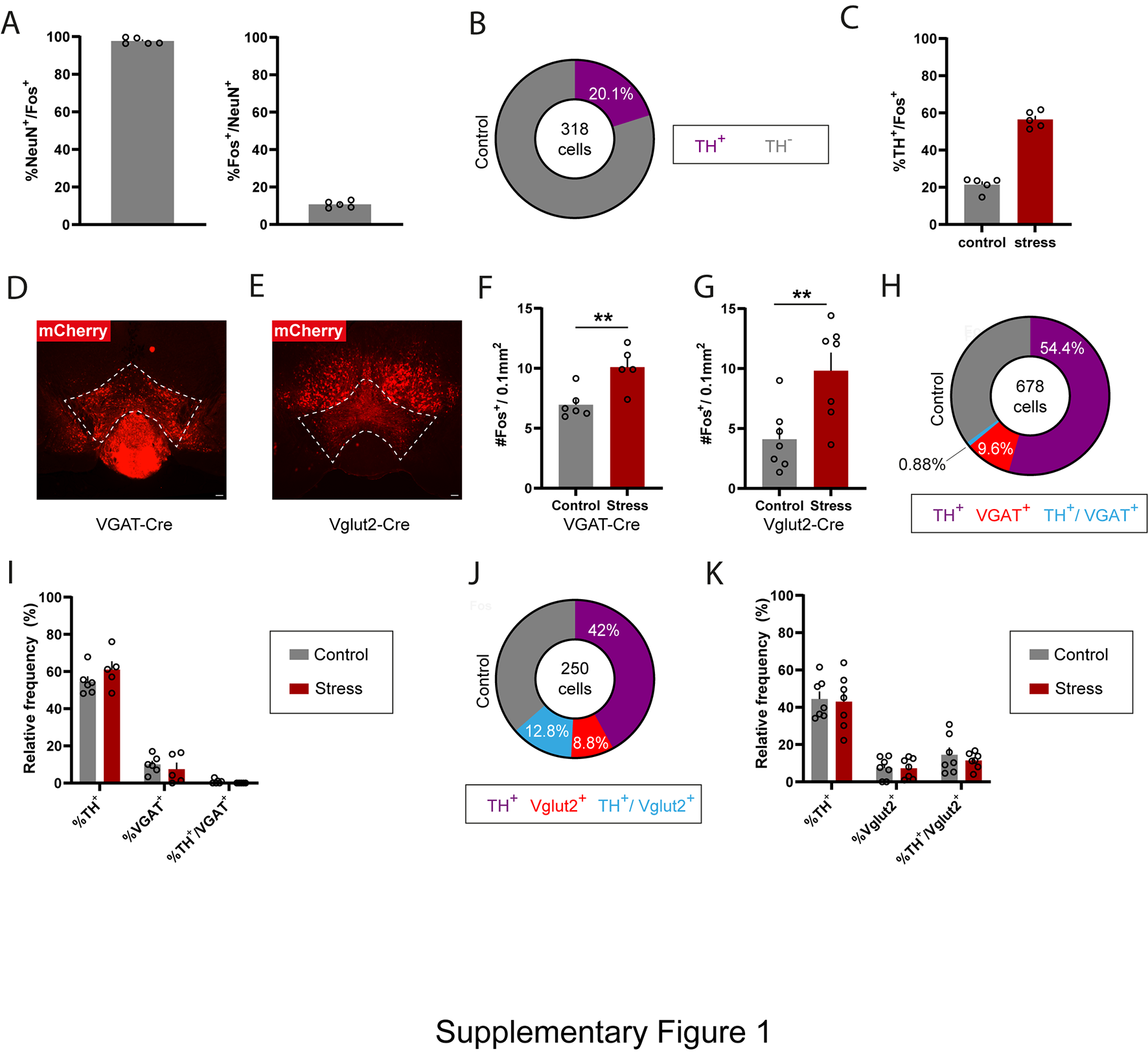

Supplement: Supplementary Figure 1 — (A) Left: Bar chart showing the proportion of NeuN+ cells within the subset of stress-implicated Fos+ cells across animals (n = 5 mice). Right: Bar chart showing the proportion of Fos+ cells out of all NeuN+ VTA cells across animals (n = 5 mice). (B) Pie chart showing the proportion of dopaminergic neurons in control-activated VTA neurons (n = 5 mice). (C) Bar chart showing the proportion of TH+ cells out of all Fos+ cells across animals (ncontrol = 5 mice, nstress = 5 mice). (D) Representative image showing distribution of GABAergic neurons in the VTA, after viral delivery of AAV-hSyn-DIO-mCherry in VGAT-Cre mice. Scale bar: 200 microns. (E) Representative image showing distribution of glutamatergic neurons in the VTA, after viral delivery of AAV-hSyn-DIO-mCherry in Vglut2-Cre mice. Scale bar: 200 microns. (F) Bar chart showing the quantification of VTA Fos+ neurons in VGAT-Cre control and stressed animals (ncontrol = 6, nstress = 5 mice). Data are presented as mean + SEM, with individual data points (cells) plotted alongside. (G) Bar chart showing the quantification of VTA Fos+ neurons in Vglut2-Cre control and stressed animals (ncontrol = 7, nstress = 7 mice). Data are presented as mean + SEM, with individual data points (cells) plotted alongside. (H) Pie chart showing the molecular identity of control-activated VTA neurons in VGAT-Cre animals (n = 6 mice; pooled data). (I) Bar chart showing the molecular identity of Fos+ cells in VGAT-Cre animals across animals (ncontrol = 6, nstress = 5 mice). (J) Pie chart showing the molecular identity of control-activated VTA neurons in Vglut2-Cre animals (n = 7 mice; pooled data). (K) Bar chart showing the molecular identity of Fos+ cells in Vglut2-Cre animals (ncontrol = 7, nstress = 7). **p < 0.01. [file Image_1.TIF]

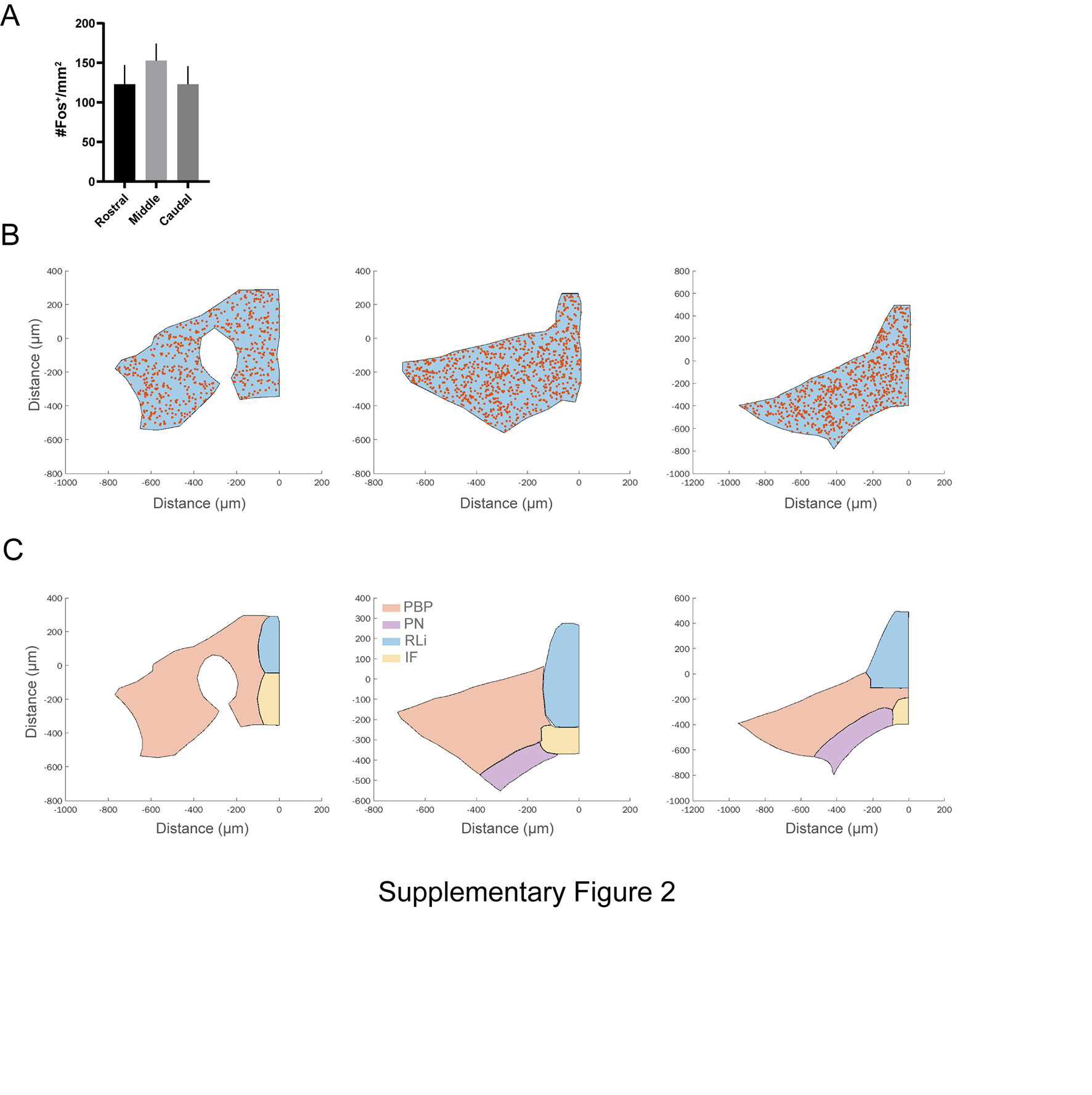

Supplement: Supplementary Figure 2 — (A) Bar chart showing the number of Fos+ neurons in different zones along the anterior-posterior axis of the VTA: rostral, middle and caudal. (B) Randomly generated (simulated) points (red) inside the geometric boundaries of the VTA (light blue area). Number of points was equal to the total number of experimentally observed Fos+ cells in the respective AP zone (nrostral = 569; nmiddle = 787; ncaudal = 712). (C) Model VTA subregion delineation, based on Paxinos mouse brain atlas. Parabrachial pigmented area (PBP; beige), paranigral nucleus (PN; violet), interfascicular nucleus (IF; yellow) and the rostral linear nucleus (RLi; blue). [file Image_2.TIF]

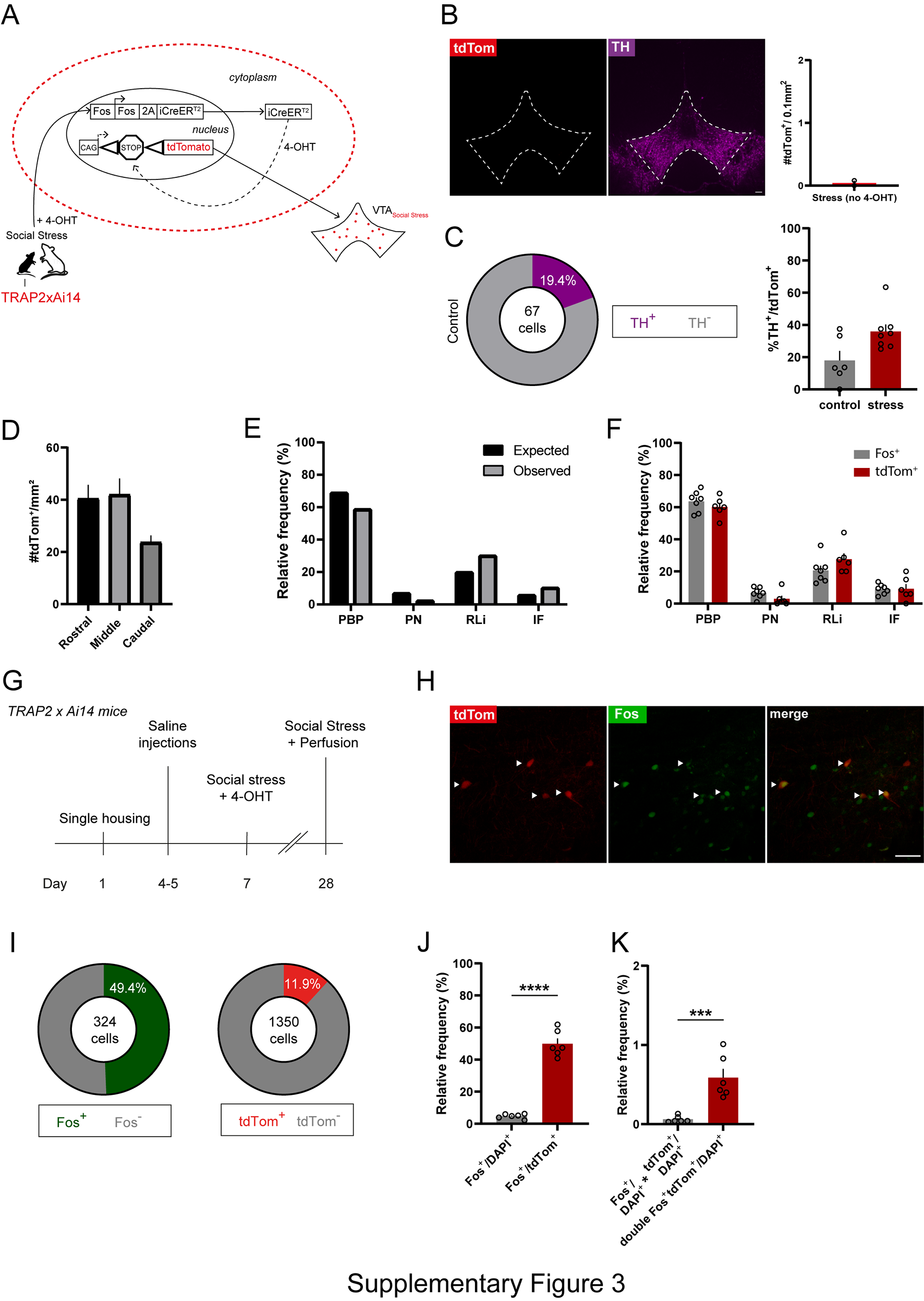

Supplement: Supplementary Figure 3 — (A) Schematic depiction of TRAP2 mechanism of action. Upon social stress, c-Fos promoter drives the expression of Fos and iCreERT2 (tamoxifen-dependent Cre). In the presence of 4-OHT, Cre translocates to the cell nucleus where it induces recombination of the fluorescent reporter tdTomato. (B) Left: Representative image of a VTA section of an animal that underwent social stress in the absence of 4-OHT injection. Scale bar: 200 microns. Right: Bar chart quantification of stress-TRAPed VTA cells in the absence of 4-OHT (n = 2 mice). (C) Left: Pie chart showing the proportion of dopaminergic cells within a control-TRAPed VTA neuronal ensemble (n = 6 TRAP2xAi14 mice that received 4-OHT 3 h after the start of a control session; pooled data). Right: Bar chart showing the proportion of TH+ cells out of TRAPed cells across animals (ncontrol = 6 mice, nstress = 8 mice). (D) Bar chart showing the number of stress-TRAPed neurons in different zones along the anterior-posterior axis of the VTA: rostral, middle and caudal across animals (n = 6 mice). (E) Proportion of observed and expected stress-TRAPed cells per VTA subregion: parabrachial pigmented area (PBP), paranigral nucleus (PN), interfascicular nucleus (IF) and the rostral linear nucleus (RLi). (F) Bar chart showing the VTA subregional distribution of acute stress implicated Fos+ and stress-TRAPed cells across animals [nFos = 7 mice, ntdTom(stress–TRAP) = 6 mice]. (G) Experimental timeline to determine the extent to which the stress-TRAPed neuronal ensemble is reactivated by a second social stressor. (H) Representative images of Fos staining in VTA slices of a mouse that underwent stress-TRAPing 3 weeks earlier. White arrowheads indicate colocalization of Fos and tdTomato. Scale bar: 50 microns. (I) Left: Pie chart showing the proportion of stress-reactivated neurons (#double Fos+ and TRAPed out of total TRAPed). Right: Pie chart showing the proportion of TRAPed neurons out of the total Fos+ population (#double TRAPed and [file Image_3.TIF]

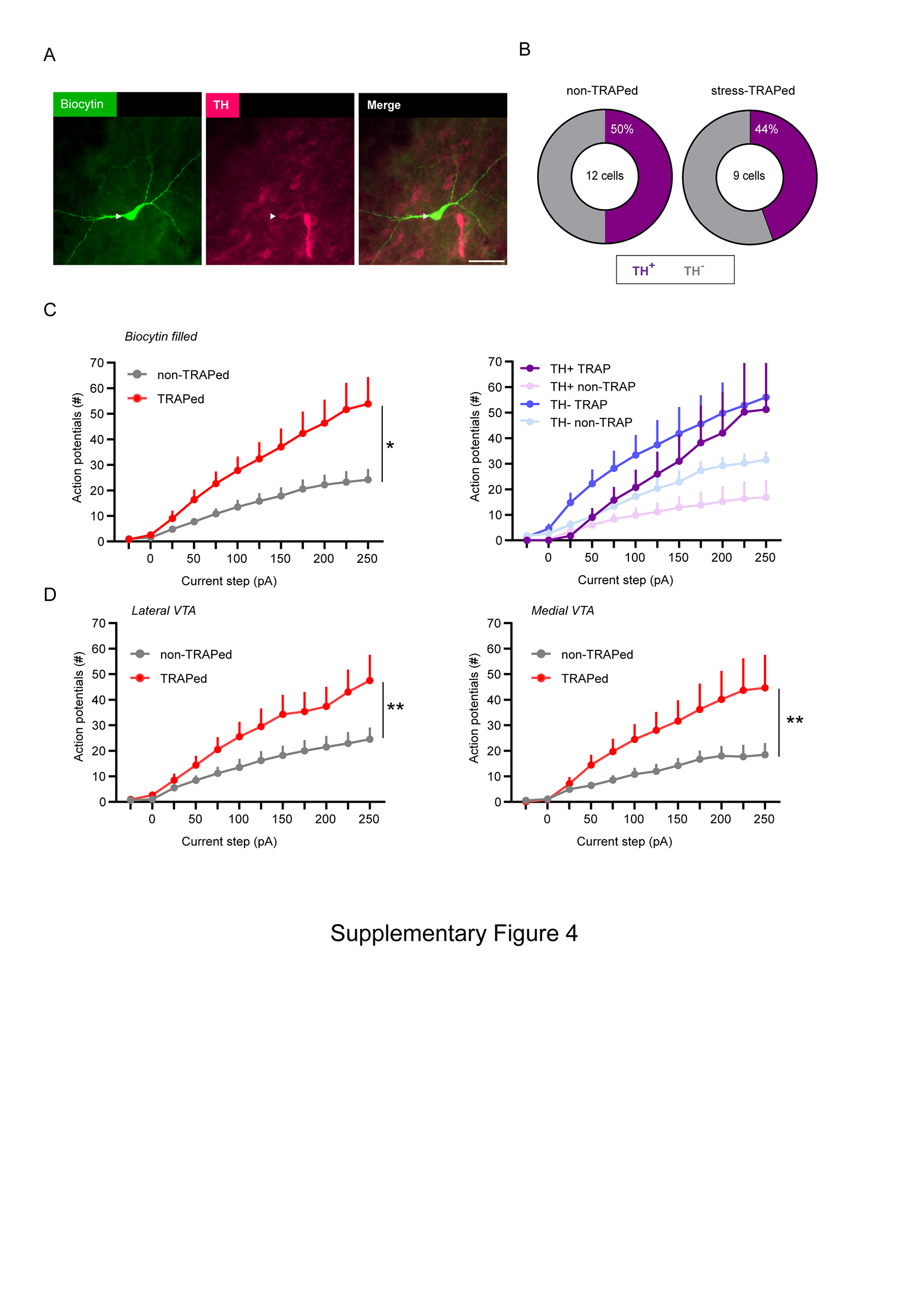

Supplement: Supplementary Figure 4 — (A) Representative image of fluorescently labeled biocytin filled neurons (green), stained for dopamine marker TH (red). White arrowhead indicates a neuron positive for biocytin and TH. Scalebar: 10 microns. (B) Pie charts of the amount of TH+ and TH– cells within the biocytin filled cellular population in non-TRAPed (n = 12 cells) and stress-TRAPed neurons (n = 9 cells). (C) Left: Line plot showing the number of action potentials fired as a function of injected current in all biocytin-labeled VTA neurons in non-TRAPed cells (n = 12 cells) versus stress-TRAPed cells (n = 9 cells). Right: Line plot showing the separate curves for TH+ non-TRAPed (n = 6) vs. stress-TRAPed (n = 4), and TH– non-TRAPed (n = 6 cells) vs. stress-TRAPed (n = 5 cells). (D) Left: Line plot showing the number of action potentials fired by non-TRAPed (n = 10 cells) and stress-TRAPed cells (n = 8 cells) in the lateral VTA. Right: Line plot showing the number of action potentials fired by non-TRAPed (n = 11 cells) and stress-TRAPed cells (n = 9 cells) in the medial VTA. *p < 0.05, **p < 0.01. [file Image_4.TIF]
